# EEG response of dexmedetomidine during drug induced sleep endoscopy

**DOI:** 10.3389/fnins.2023.1144141

**Published:** 2023-07-14

**Authors:** Lichy Han, David R. Drover, Marianne C. Chen, Amit R. Saxena, Sarah L. Eagleman, Vladimir Nekhendzy, Angelica Pritchard, Robson Capasso

**Affiliations:** ^1^Department of Anesthesiology, Perioperative and Pain Medicine, Stanford University, Stanford, CA, United States; ^2^Department of Neurology and Neurological Sciences, Stanford University, Stanford, CA, United States; ^3^Department of Otolaryngology, Head and Neck Surgery, Stanford University, Stanford, CA, United States

**Keywords:** dexmedetomidine, level of sedation, electroencephalography, drug induced sleep endoscopy, pharmacology

## Abstract

**Introduction:**

Dexmedetomidine is one of the anesthetics of choice for drug induced sleep endoscopy (DISE), with advantages including limited respiratory depression, analgesia, and decreased incidence of emergence delirium. However, challenges with determining sedation levels and prolonged recovery have limited its usage. An improved understanding of the effect of dexmedetomidine on the level of sedation and the corresponding electroencephalographic (EEG) changes could help overcome these barriers.

**Methods:**

Fifty-one patients received dexmedetomidine sedation with Richmond Agitation-Sedation Scale (RASS) score assessment and continuous EEG monitoring via SedLine for DISE. We constructed a pharmacokinetic model to determine continuous dexmedetomidine blood concentration. From the SedLine, we extracted the patient state index (PSI), and from the EEG we calculated the spectral edge frequency 95% (SEF95) and the correlation dimension (CD), a type of fractal dimension used to assess the complexity of a system. These metrics were subsequently compared against one another and with the dexmedetomidine concentration.

**Results:**

Our pharmacokinetic model yielded a two-compartment model with volumes of 51.8 L and 106.2 L, with clearances of 69.5 and 168.9 L/h, respectively, and a time to effect of 9 min, similar to prior studies. Based on this model, decreasing RASS score, SEF95, CD, and PSI were all significantly associated with increasing dexmedetomidine concentration (*p* < 0.001, *p* = 0.006, *p* < 0.001 respectively). The CD, SEF95, and PSI better captured the effects of increasing dexmedetomidine concentration as compared to the RASS score. Simulating dexmedetomidine concentration based on titration to target levels derived from CD and PSI confirmed commonly used dexmedetomidine infusion dosages.

**Conclusion:**

Dexmedetomidine use for DISE confirmed previous pharmacokinetic models seen with dexmedetomidine. Complex EEG metrics such as PSI and CD, as compared to RASS score and SEF95, better captured changes in brain state from dexmedetomidine and have potential to improve the monitoring of dexmedetomidine sedation.

## 1. Introduction

Dexmedetomidine is a sedative with many advantageous characteristics, including limited respiratory depression, anxiolysis, analgesia, and is thought to more closely resemble natural sleep compared to other anesthetic agents (Huupponen et al., 2008; Akeju et al., 2018). Due to these qualities, it is one of the anesthetic options for drug induced sleep endoscopy (DISE) (Capasso et al., 2016), whereby the patient is sedated in an attempt to mimic natural sleep to evaluate for anatomic and physiologic causes of upper airway obstruction. The use of dexmedetomidine has been limited, however, in part by the inconsistency in its response, prompting the need for improved sedation assessment with its use (Holliday et al., 2014). The Richmond Agitation-Sedation Scale (RASS) (Sessler et al., 2002) is a popular tool for sedation assessment; however is subjective and does not capture the continuum of brain states as it categorizes patients into one of 10 states. While the addition of electroencephalogram (EEG) monitoring can overcome some of these limitations, the EEG signatures of dexmedetomidine are not well characterized. The anesthetic effect of dexmedetomidine is different from other popular anesthetics such as propofol and fluorinated hydrocarbons in that it induces a more natural sleep-like EEG pattern primarily generating EEG slow waves without a significant predominant alpha frequency (Nelson et al., 2003; Akeju et al., 2014).

Previous EEG markers, such as the bispectral index, have been unable to determine differences elicited with dexmedetomidine (Kaskinoro et al., 2011), thus motivating the discovery of novel EEG markers associated with changes in brain state seen with dexmedetomidine sedation. Other commonly used EEG monitoring metrics, such as the patient state index (PSI), have not been well described with dexmedetomidine sedation. Characterizing EEG complexity *via* nonlinear dynamical changes that occur under anesthesia has been shown to reveal new insights into the activity of the unconscious brain (Natarajan et al., 2004; Stam, 2005; Eagleman et al., 2019). One complexity measure that significantly differs among arousal states is correlation dimension (CD) (Grassberger and Procaccia, 1983), a type of fractal dimension frequently applied to time series data, including EEG.

We performed a pharmacokinetic and pharmacodynamic analysis to assess the effects of dexmedetomidine on the EEG and corresponding brain state. In doing so, we aimed to characterize and compare the effects of dexmedetomidine using spectral analysis, complexity analysis, in addition to evaluating existing metrics such as PSI and the RASS score. Comparison of these metrics will enable us to identify markers to ultimately improve sedation monitoring for procedural sedation under dexmedetomidine.

## 2. Materials and methods

### 2.1. Study design and data collection

Fifty-one subjects were recruited into this prospective observational data collection study. All subjects were considered in a consecutive manner for inclusion if they were more than 18 years of age and American Society of Anesthesiologists (ASA) physical status I, II, or III who were scheduled for an elective drug induced sleep endoscopy (DISE) study for the diagnosis and possible treatment of obstructive sleep apnea. Subjects with ASA physical status greater than III, or who could not complete all data collection, or who had any contraindication to any study procedures were excluded from the study upon screening. The protocol was approved by the Stanford University Research Compliance Office (Institutional Review Board Protocol #34322) and all patients were provided study information and signed a written informed consent.

Subjects had 2 peripheral intravenous catheters placed, one in each arm; one intravenous catheter was for infusion of dexmedetomidine and another was placed in the opposite arm for draw of venous blood collection to later determine dexmedetomidine plasma concentrations. Patient monitoring included 5-lead electrocardiogram, non-invasive blood pressure, pulse oximeter, respiratory rate acoustic monitoring (RRa©, Masimo, Irvine, CA), and electroencephalography (EEG) (SedLine©, Masimo, Irvine, CA). Patient information including pulse oximetry, RRa, and EEG were downloaded directly to a laptop for future data analysis. Patient state index (PSI) was recorded from the SedLine data, as a marker of sedation from 0 (most sedated) to 100 (least sedated). The subjects did not receive preoperative sedation or supplemental oxygen as it was deemed to interfere with the DISE procedure results.

Once all data connections were confirmed, the patients were placed in a supine position, the lights in the operating room were dimmed, and the patients were asked to lie quietly with their eyes closed. Lidocaine jelly was applied to the nares, and then a video scope was placed in the nose to record the results of the endoscopy prior to initiation of dexmedetomidine to minimize changes in stimulation during the study. After a baseline period of EEG recording of at least 3 min, a loading infusion of dexmedetomidine was begun at 1 to 1.5 mcg/kg over 10–15 min followed by a maintenance infusion of 1–1.5 mcg/kg/h until completion of the DISE. All dexmedetomidine dosages were infused by an infusion pump and all changes and dosages were recorded.

Blood was collected from the indwelling IV catheter every 15 min after start of dexmedetomidine for up to 2 h or until the patient was discharged home. Blood was processed and stored frozen at −70° C until ready for analysis. Dexmedetomidine concentrations were performed by liquid chromatography with tandem mass spectrometry (LC–MS/MS) using the AB Sciex API 4000™ system (SCIEX, Framingham, MA) by iC42 Clinical Research and Development in Aurora, CO. This laboratory is accredited by the Clinical Laboratory Improvement Amendments (CLIA) and by the College of American Pathologists (CAP). The pharmacokinetics were determined from the blood sample results using the Phoenix software (Certara©, Princeton, NJ).

Richmond Agitation-Sedation Scale (RASS) score (Sessler et al., 2002) was used as a measure of subject level of sedation. The RASS score was recorded at baseline (pre-dexmedetomidine sedation), and then during the dexmedetomidine infusion every 5 min until the end of the DISE procedure. For those patients who did not continue to a general anesthesia procedure after DISE, the RASS score was recorded every 15 min until the end of dexmedetomidine blood sampling.

### 2.2. EEG preprocessing

The EEG recordings captured consisted of 5 channels (F7, Fp1, Fp2, F8, and reference, AFz) with a sampling frequency of 250 Hz for the duration of the procedure and during recovery. EEG data were preprocessed by detrending, followed by a low pass filter of 50 Hz and high pass filter of 1 Hz. We then performed a 2-level wavelet decomposition to further remove noise and artifacts from the data. Following preprocessing, EEG data were split into 30 s intervals, each corresponding to a simulated concentration of dexmedetomidine. Intervals were rejected if the amplitude exceeded 100 μV, if the activity was lower than 0.5 μV, or if amplitude changes exceeded 100 μV within 100 ms. If additional sedative agents were given, all intervals after that point in time were excluded from analysis.

### 2.3. Pharmacokinetic model construction and simulations

We used a pharmacokinetic model constructed using Phoenix 8.3 (Certara, Princeton, NJ) to simulate the blood concentration of dexmedetomidine. We constructed models with one to three compartments, tested additive and log-additive error, and included covariates age, weight, body mass index (BMI), and fat free mass (FFM). The model with the best performance based on the Akaike information criterion (AIC) was then used to calculate simulated blood concentration levels at 30 s intervals for downstream analysis. Lower AIC values correspond to better model performance.

We analyzed the time to effect of dexmedetomidine by creating delayed linear mixed models, with the patient as a random effect. The time to effect was chosen as the delay time that maximized the relationship between the dexmedetomidine concentration and RASS score with the highest coefficient and lowest value of *p*.

### 2.4. Spectral analysis

We calculated the spectral edge frequency (SEF95), defined as the frequency below which 95 percent of the total EEG power is contained, for 30-s intervals of EEG data for all channels. The SEF95 values were then averaged across all channels and compared to the RASS score, average correlation dimension (as defined below), and dexmedetomidine blood concentration.

Using channel specific data, we then analyzed the power spectral density over the course of the procedure based on the dexmedetomidine concentration, RASS score, SEF95, and correlation dimension (CD). For dexmedetomidine concentration, SEF95, and CD, we divided the data into quartiles based on each calculated measure and averaged the power spectral density for each quartile for each channel. For RASS score, we examined the power spectral density of all 6 scores separately. Analyzing the spectra in this manner enabled us to visualize changes in the EEG spectrum at different levels of sedation.

We determined the significance between power spectra using the two-group spectrum test from the Chronux toolbox.^1^ In accordance with previous studies, a threshold of *p* = 0.001 was used to determine significance. Akeju et al. (2018) *p*-values were adjusted for multiple hypothesis testing using the Bonferroni correction.

### 2.5. Complexity analysis

Correlation dimension was calculated using a dimension embedding delay of 8 milliseconds and 5 dimensions. As described by Grassberger and Procaccia (1983) correlation dimension is defined as:


$$C\left(r\right)=\lim_{N\to \infty }\frac{\left\{\#\mathrm{pairs}\;\left(i,j\right)\;\mathrm{whose distance}\;|X_{i}-X_{j}|<r\right\}}{N^{2}}$$

$$\mathrm{where}\;C\left(r\right)\sim r^{D}$$

where $C\left(r\right)$ is the correlation integral, N is the number of points, r is the neighborhood radius, and D is the correlation dimension. The bounds on the neighborhood radius to determine the linear portion of the logC versus logr plot were chosen as the 10th and 90th percentile range of the first derivative. All signal processing and correlation dimension analysis was done using Matlab R2021a (MathWorks, Natick, MA).

### 2.6. Comparing EEG markers

We analyzed the relationship among the dexmedetomidine level, RASS score, spectral characteristics, and CD using R 3.6.1 (R Core Team, Vienna, Austria). We constructed cumulative link mixed models to analyze the relationship between RASS score and other variables, representing the RASS score as an ordinal variable, with the patient number as a random effect. For continuous variables such as SEF95, CD, and PSI, we used exponential decay models to analyze the relationship between them and the dexmedetomidine blood level. Significance was based on a threshold of *p* = 0.05.

## 3. Results

### 3.1. Patient and data characteristics

Patient demographics for our patients are shown in Table 1. Overall, our patient population consisted of mostly males (*N* = 40) with a high body mass index, of which the majority were physical status 2 under the ASA classification. One patient was given glycopyrrolate and ephedrine during the procedure, and one other patient was given glycopyrrolate only. No other patients required hemodynamic support. The measured dexmedetomidine concentrations over time for our patients is shown in Supplementary Figure S1.

**Table 1:** Characteristics of the 46 subjects that completed the study.

	Age	Height	Weight	BMI	ASA	FFM
	(year)	(cm)	(kg)	(kg/m*m)		(kg)
Mean	54.1	175.4	90.3	29.3	2.1	64.4
SD	11.9	9.5	17.3	4.4	0.5	9
Min	26	153.7	54.6	18.9	1	47.1
Max	82	193	135	39.1	3	86.2

cm, centimeters; kg, kilograms; m, meters; BMI, body mass index; FFM, fat free mass.

**Figure 1:**
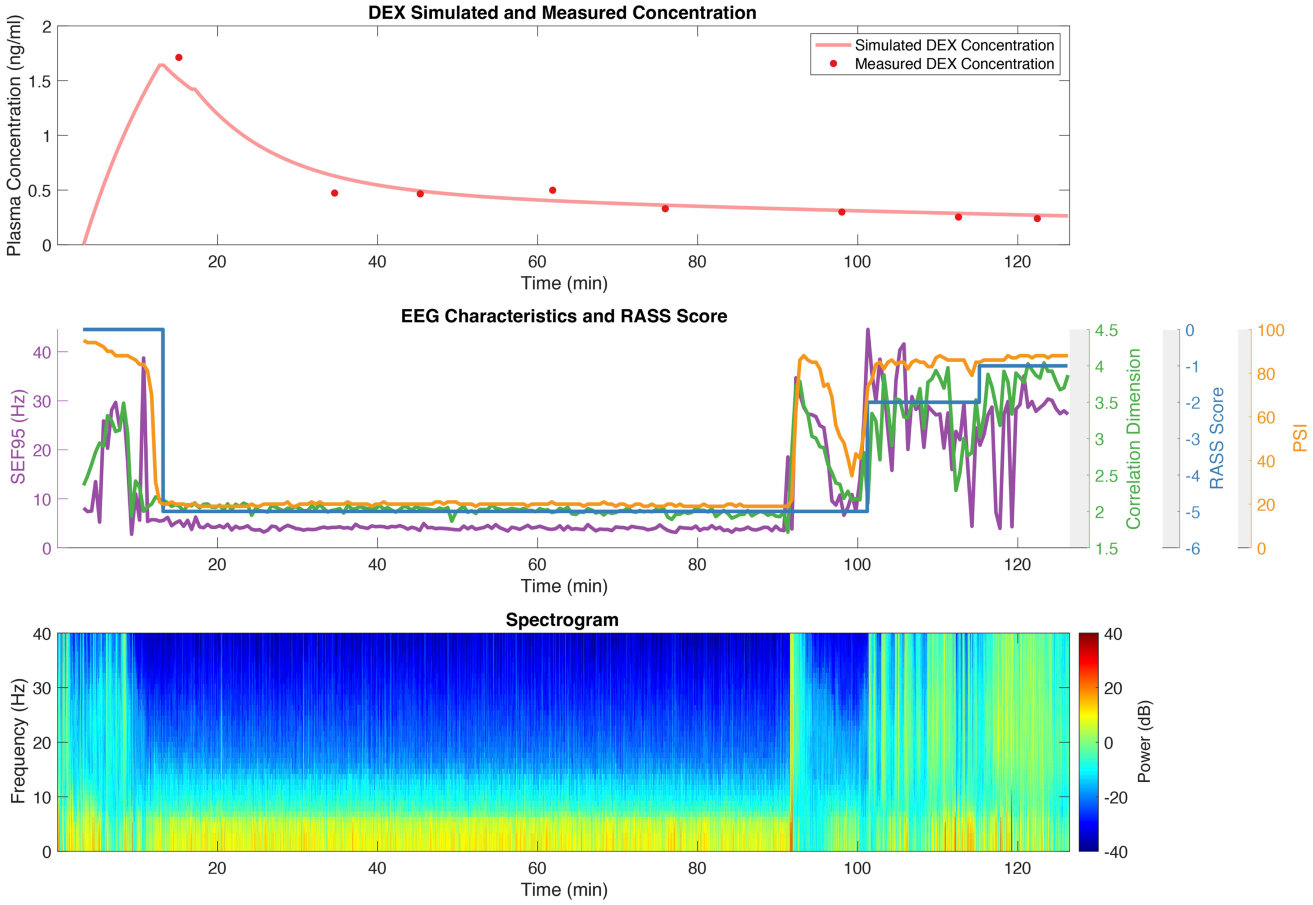
Dexmedetomidine (DEX) concentrations; RASS scores; and SEF95, CD, and PSI derived from EEG for a representative patient. The top panel depicts the measured dexmedetomidine concentration with red dots, and the simulated concentration in pink derived from our pharmacokinetic model. The middle panel shows the RASS score (blue), CD (green), SEF95 (purple), and PSI (orange) throughout the procedure. The bottom panel is the spectrogram of the processed EEG data.

An example of the dexmedetomidine concentrations, RASS scores, calculated EEG features, and EEG spectrum over the course of a case is shown in Figure 1 for a representative patient. The top panel shows the measured dexmedetomidine samples (red dots), with the continuous simulated level based on our pharmacokinetic model shown in pink. The middle panel depicts the RASS score, SEF95, CD, and PSI throughout the procedure. A spectrogram, after cleaning and preprocessing, is shown in the bottom panel, from which the SEF95 and CD were calculated.

### 3.2. Pharmacokinetic model

A two-compartment model with an additive error was the best fit for our data (AIC = −425.1) as compared to a one-compartment model (AIC = −88.4) or three-compartment model (AIC = −239.0). Adding single covariates and combinations of covariates did decrease the AIC however the improvement in the model was not statistically significant. Thus, a two-compartment model was used to model dexmedetomidine concentration, with compartment volumes of 51.8 L and 106.2 L and clearances of 69.5 L/h and 168.9 L/h, respectively. Based on our model, the relationship between the actual and predicted dexmedetomidine blood concentrations is depicted in Supplementary Figure S2.

In determining the time to effect, we found that 9 min optimized the relationship between the dexmedetomidine blood concentration and the RASS score, with a t-value of −10.994 corresponding to a value of *p*-value, of <2e-16.

### 3.3. Spectral analysis By quartile

**Figure 2:**
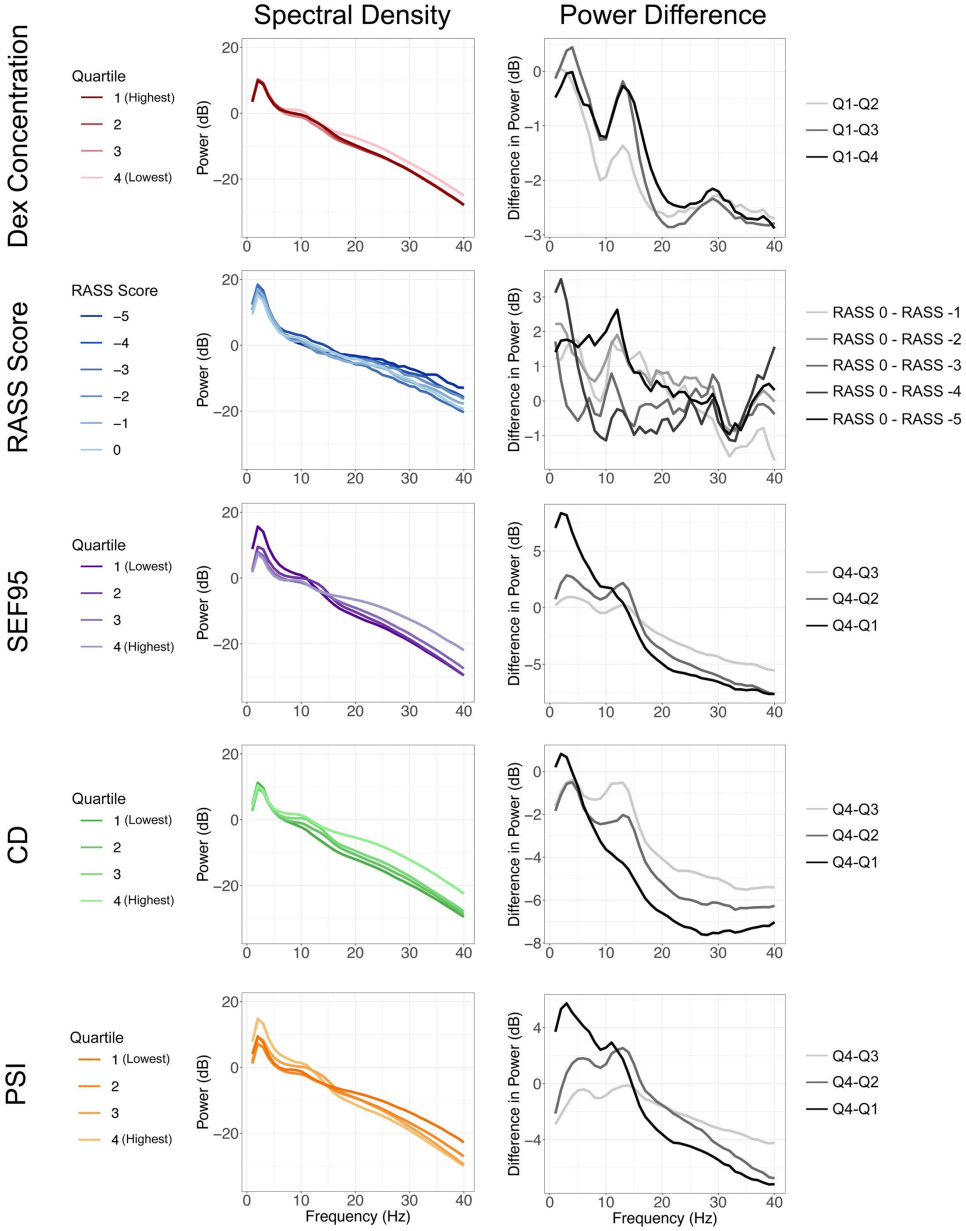
Spectral density plots (left column) stratified by dexmedetomidine concentration, RASS score, SEF95, CD, and PSI. The varying shades of each color represent increasing sedation from light to dark. Difference of each quartile from the quartile representing the least sedation (right column). Changes in power seen with increasing sedation are represented from light to dark.

After preprocessing, we analyzed a total of 3,257 thirty-second EEG segments. The overall power density spectra for different groups of dexmedetomidine concentration, RASS score, SEF95, and CD for one channel are depicted in Figure 2 (left column). The differences between the lowest level of sedation and all other groups are also shown to highlight changes in different frequency bands of interest (Figure 2, right column). Increasing blood levels of dexmedetomidine resulted in a significant decrease in power across the entire spectrum, with relative preservation in the 10–15 Hz band, consistent with spindle oscillations. These findings were also significant for SEF95 and CD. For SEF95 and CD, at the lowest quartile, there is a shift to greater power in the delta band (1–5 Hz), with a reduction of spindle oscillations. Notably, there was no clear trend between sedation level as measured by the RASS score and changes in the EEG (Figure 2). Varying RASS score comparisons fail to capture even a global decrease in power, which was seen with increasing dexmedetomidine concentration. In the 10–15 Hz region, only the difference between a RASS score of 0 and − 5 was significant. Overall, RASS score was least able to capture the brain state changes seen with dexmedetomidine, suggesting other metrics for titrating dexmedetomidine sedation may be of greater value.

### 3.4. Comparing EEG markers

**Figure 3:**
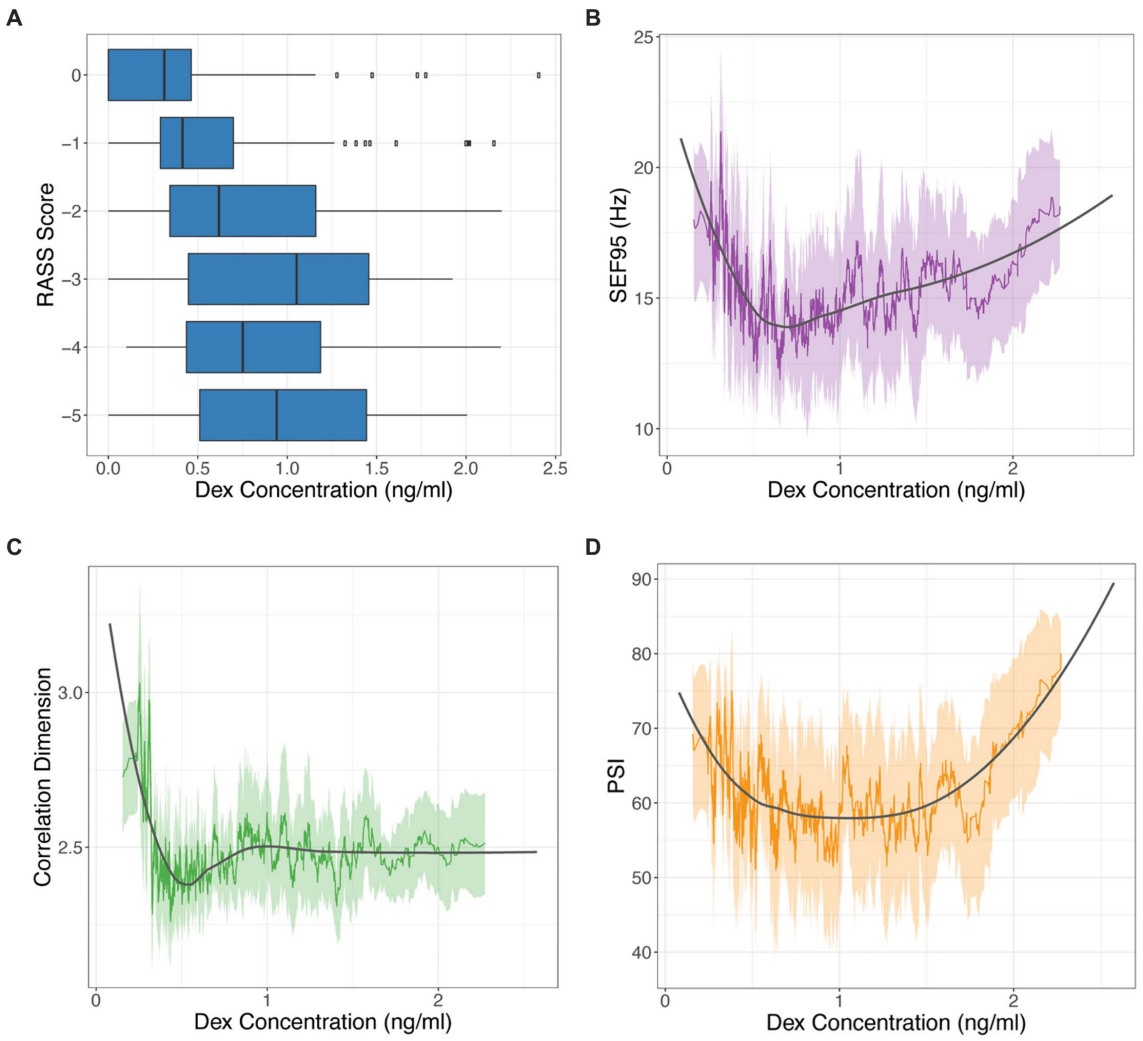
RASS score **(A)**, SEF95 **(B)**, CD **(C)**, and PSI **(D)** versus dexmedetomidine (DEX) concentration. SEF95, CD, and PSI are depicted as rolling mean in the colored solid line, the standard deviation in the shaded area, with local polynomial regression (LOESS) fitting shown in black.

When comparing our markers and dexmedetomidine levels, we observed decreasing RASS scores with increasing dexmedetomidine concentrations (Figure 3A), which we had optimized for when calculating our time delay (*p* < 0.001). SEF95, CD, and PSI all significantly decrease with increasing dexmedetomidine concentration (*p* = 0.006, *p* < 0.001, *p* < 0.001 respectively) (Figures 3B,C). A decrease in CD from 3–4 to 2–2.5 is seen with increasing dexmedetomidine levels, suggesting that the correlation dimension captures a decrease in brain signal complexity under dexmedetomidine (Figure 3C). A similar trend is seen with SEF95 and PSI (Figures 3B,D), however notably at very high concentrations, they both exhibit an upwards trajectory.

**Figure 4:**
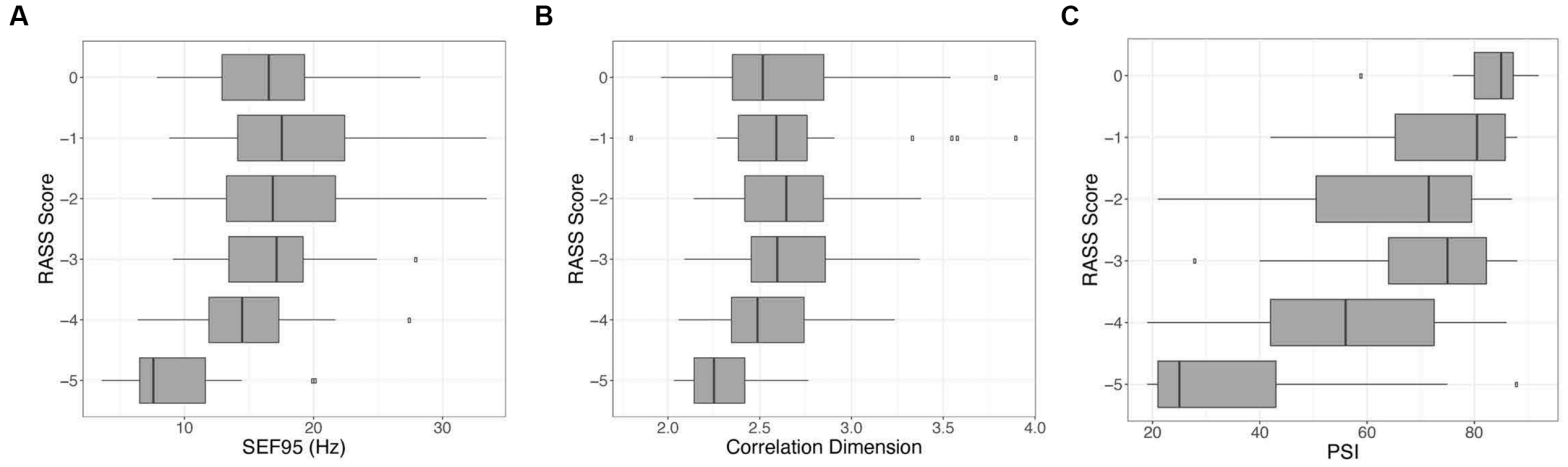
Visualization of the relationship between the RASS score versus SEF95 **(A)**, CD **(B)**, and PSI **(C)**. Decreasing RASS scores are significantly associated with decreasing SEF95, CD, and PSI, particularly for deeper levels of sedation. The boxes represent the median with interquartile range, whereas the whiskers represent the remainder of the range.

When comparing these markers among themselves, CD and SEF95 are significantly correlated (*p* < 0.001, *r* = 0.515). Notably, there are a subset of points that exhibit a high SEF95 but a low CD (data not shown). PSI is most strongly correlated with SEF95 (*p* < 0.001, *r* = 0.631). There is a significant decrease in the SEF95, CD, and PSI with decreasing RASS score (*p* < 0.001, *p* = 0.02, *p* < 0.001 respectively) (Figure 4). The decrease is most notable for deeper levels of sedation corresponding to a RASS score of −4 and − 5, suggesting that these metrics may be most beneficial for deeper levels of sedation.

### 3.5. Simulated dosing regimens

**Figure 5:**
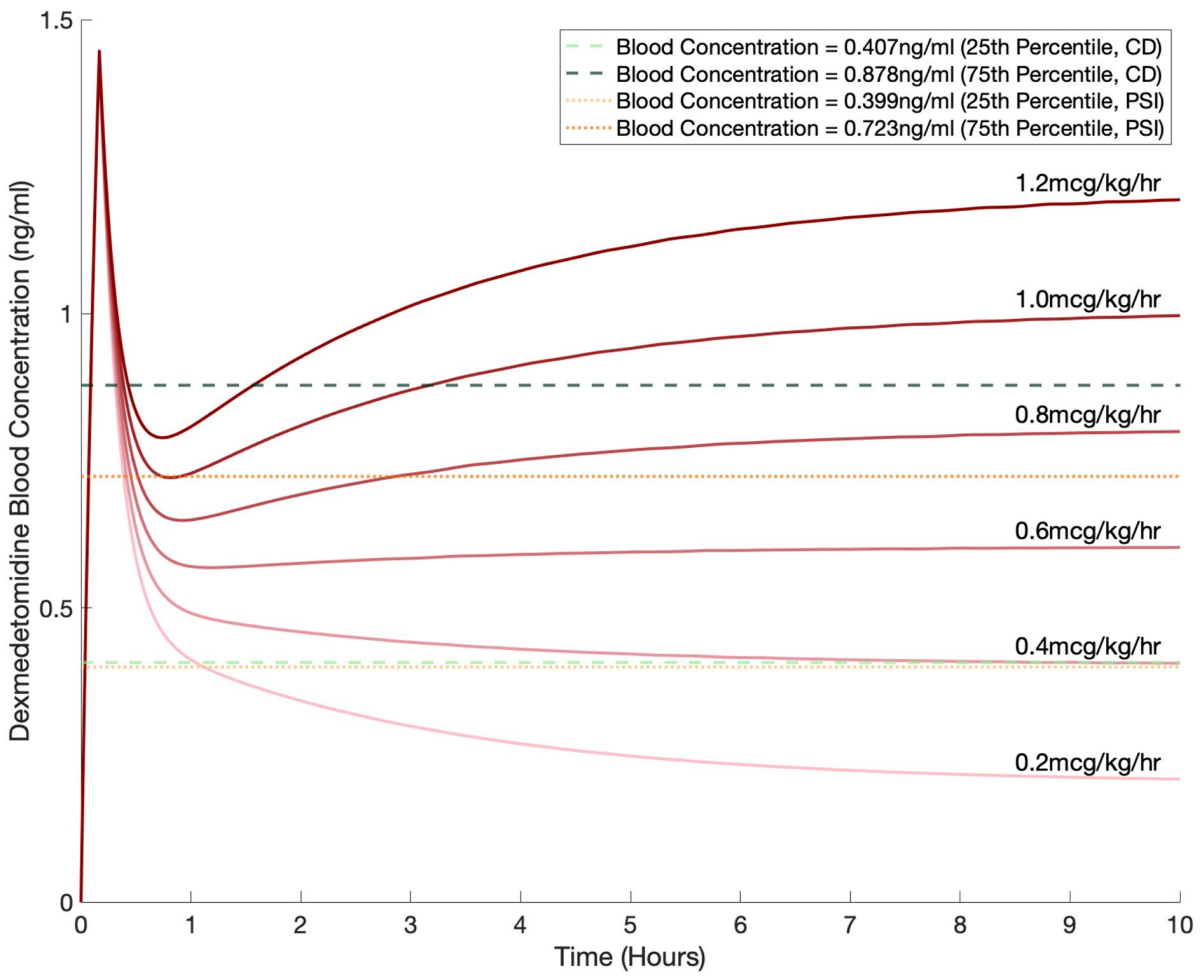
Simulated blood dexmedetomidine concentrations based on commonly used regimens of 0.2, 0.4, 0.6, 0.8, 1, and 1.2 mcg/kg/h infusion after a 1.5 mcg/kg bolus for a 70 kg patient. Blood concentration levels of 0.407 ng/mL and 0.878 ng/mL, selected based on the correlation dimension analysis, are shown by the horizontal dashed green lines. Blood concentration levels of 0.399 ng/mL and 0.723 ng/mL, selected based on the PSI, are shown by the horizontal dotted orange lines.

We used our pharmacokinetic model to simulate dexmedetomidine dosing with a 1.5 mcg/kg bolus over 10 min followed by an infusion for a 70 kg patient (Figure 5). For the infusions, we simulated commonly used dosages of 0.2, 0.4, 0.6, 0.8, 1.0, and 1.2 mcg/kg/h. Based on the correlation dimension analysis (Figure 3C) and the local polynomial regression, we selected a CD of 2.5 as our marker threshold. Based on our data, this corresponded to a dexmedetomidine concentration with a median of 0.538 ng/mL, with an interquartile range of 0.407 ng/mL to 0.878 ng/mL (Figure 5). We also selected thresholds based on PSI values of 25–50, which are generally considered to correspond to an optimal range of sedation. This corresponded to a dexmedetomidine concentration with a median of 0.500 ng/mL, with an interquartile range of 0.399 ng/mL to 0.723 ng/mL. The lower dexmedetomidine infusion range leveled out to a steady state concentration that fell between the limits we derived.

## 4. Discussion

In this work, we present a pharmacokinetic and pharmacodynamic analysis of dexmedetomidine and its effects on the EEG and level of sedation. For our pharmacokinetic analysis, we derived a two-compartment model as our optimal model. This aligns with multiple previously published models, which have analyzed dexmedetomidine pharmacokinetics in various settings, including in the postoperative setting and in the intensive care unit (Talke et al., 1997; Dutta et al., 2000; Venn et al., 2002; Iirola et al., 2012; Cortínez et al., 2015). Our model, which yielded compartment volumes of 51.8 L and 106.2 L with clearances of 69.5 L/h and 168.9 L/h, is similar to models published by previous studies in both the perioperative and intensive care unit settings, thus providing a solid foundation for our subsequent pharmacodynamic analysis (Talke et al., 1997; Dutta et al., 2000; Venn et al., 2002; Iirola et al., 2012; Cortínez et al., 2015; Yan et al., 2021). Using our model, we derived a time to effect of 9 min by optimizing the relationship between the dexmedetomidine concentration and RASS score. This is in agreement with previous studies, which have described the onset time of dexmedetomidine as 5–10 min, with a peak effect at 15 min (Naaz and Ozair, 2014).

Our patient population consisted of mostly healthy male Caucasians undergoing DISE, so it is possible that patients from a different population may have different pharmacokinetic properties, which would limit the application of our model. However, our model shows similar results to those from a broad range of pathophysiology, suggesting the applicability of our model to different populations. Furthermore, the latter study in critically ill patients by Yan et al. did not find any significant pathophysiologic covariates (Yan et al., 2021), and similarly, none of the demographic covariates significantly improved our pharmacokinetic model.

Under dexmedetomidine sedation, the overall power of the EEG decreased with increasing dexmedetomidine concentration, with preservation of power in spindle oscillations (Figure 2). This is concordant with previous studies, which have described an increase in power between 2 and 15 Hz, an overall decrease in power in the 20 to 40 Hz range, and spindle oscillations between 12 and 16 Hz (Huupponen et al., 2008; Akeju et al., 2014, 2018; Xi et al., 2018; Seo et al., 2021). These changes seen with rising dexmedetomidine concentration are significantly captured by SEF95, CD, and PSI. The RASS score, despite being the gold standard of sedation assessment, showed minimal difference in the spectra at different dexmedetomidine levels, and was unable to significantly capture changes in the brain state seen with increasing dexmedetomidine concentration. This likely reflects the sensitivity of the RASS score to user interpretation and the stimulus required to perform the assessment. By incorporating additional EEG markers of sedation, we may be able to better titrate the level of dexmedetomidine required to elicit a desired brain state.

Comparing RASS score, SEF95, CD, and PSI directly to dexmedetomidine concentration yielded significant relationships between all four markers and dexmedetomidine (Figure 3). Optimizing the relationship of the RASS score to the dexmedetomidine level still resulted in residual overlap between different RASS scores, particularly for deeper levels of sedation. By contrast, the EEG markers show a clear dose dependent decrease with an inflection point while still maintaining a significant association with the RASS score. For CD, the value with increasing dexmedetomidine concentration stays stable around 2.5. For SEF95 and PSI, an upsloping is noted at higher concentrations, and greater variability is seen with these metrics. Given that the PSI is significantly correlated with SEF95 more than CD, the upsloping at higher concentrations may be predominantly driven by changes in spectral markers captured by the SEF95. These divergences are likely due to the susceptibility of SEF95 to noise and motion artifacts from higher frequency non-EEG signals despite thorough preprocessing. Unfortunately, artifacts continue to pose a challenge in working with EEG data, particularly for procedures without paralysis or general anesthesia. Such points can also be seen in our patient example during induction and emergence, where SEF95 exhibits more variability and captures more artifact compared to CD (Figure 1), whereas PSI employs smoothing and electromyography (EMG) rejection techniques, thus decreasing its susceptibility to artifact.

Given the ability of CD and PSI to capture EEG changes of dexmedetomidine sedation and its robustness compared to SEF95 and the RASS score, we selected these two metrics to guide our dosing simulations. Dosing based on CD and PSI yielded dexmedetomidine concentrations of 0.407 to 0.878 ng/mL and 0.399 to 0.723 ng/mL respectively, similar to previously published studies (Ebert et al., 2000; Kim et al., 2020). Per the package insert for dexmedetomidine, for sedation in the intensive care unit, dexmedetomidine infusions are dosed between 0.2–0.7 mcg/kg/h, and for procedural sedation, up to 1 mcg/kg/h. Based on our simulations, these common infusion rates would yield adequate sedation based on spectral characteristics for the majority of patients.

Overall, CD and PSI best captured brain state changes seen with dexmedetomidine sedation. CD excelled at reflecting changes seen with rising dexmedetomidine concentration but appeared to exhibit a ceiling effect, whereas PSI had the most significant relationship with the RASS score. Both metrics were able to capture brain state changes corresponding to increased dexmedetomidine dose, showed decreased sensitivity to observer variability and artifact, and corresponded to currently used infusion dosages in pharmacokinetic simulations. Further investigation on the ability of complexity metrics such as CD to discern sedation level for dexmedetomidine and other sedatives is merited, particularly for deeper levels of sedation. With success, complexity metrics such as CD have the potential to augment existing metrics such as the PSI and the RASS score to help guide individualized and continuous titration of dexmedetomidine dosing.

## Data availability statement

The datasets presented in this article are not readily available because the EEG data in this study are in a proprietary format and are thus not readily available. Requests to access the datasets should be directed to LH, lhan2@stanford.edu.

## Ethics statement

The studies involving human participants were reviewed and approved by the Stanford University Institutional Review Board. The patients/participants provided their written informed consent to participate in this study.

## Author contributions

LH designed and performed the data analysis, created the figures, and wrote the majority of the manuscript. DD helped conceive the study, collect data, advised methods and analysis, and contributed to writing and editing the manuscript. MC, VN, and RC contributed to developing the study protocol and collected the data. AS helped to collect data for this study. SE collected the data, advised methods and analysis, and edited the manuscript. AP collected and analyzed the data. All authors contributed to the article and approved the submitted version.

## Funding

This study was supported by the NIH NIGMS (SE, grant number 1K99GM140215). Stanford University received funding from the Masimo, Inc. through a research grant. The funder was not involved in the study design, collection, analysis, interpretation of data, the writing of this article or the decision to submit it for publication.

## Conflict of interest

DD was a consultant for Masimo Inc. RC was a consultant for SAB – Bryte LLC and Invicta Medical.

The remaining authors declare that the research was conducted in the absence of any commercial or financial relationships that could be construed as a potential conflict of interest.

## Publisher’s note

All claims expressed in this article are solely those of the authors and do not necessarily represent those of their affiliated organizations, or those of the publisher, the editors and the reviewers. Any product that may be evaluated in this article, or claim that may be made by its manufacturer, is not guaranteed or endorsed by the publisher.

## Footnotes

^1^http://chronux.org/
