# Supplementary material for: EEG response of dexmedetomidine during drug induced sleep endoscopy
Source: Front Neurosci. 2023 Jul 14;17:1144141. doi: 10.3389/fnins.2023.1144141 (PMC10375416; doi:10.3389/fnins.2023.1144141)
Supplement: Supplementary file 1 [file Data_Sheet_1.PDF]

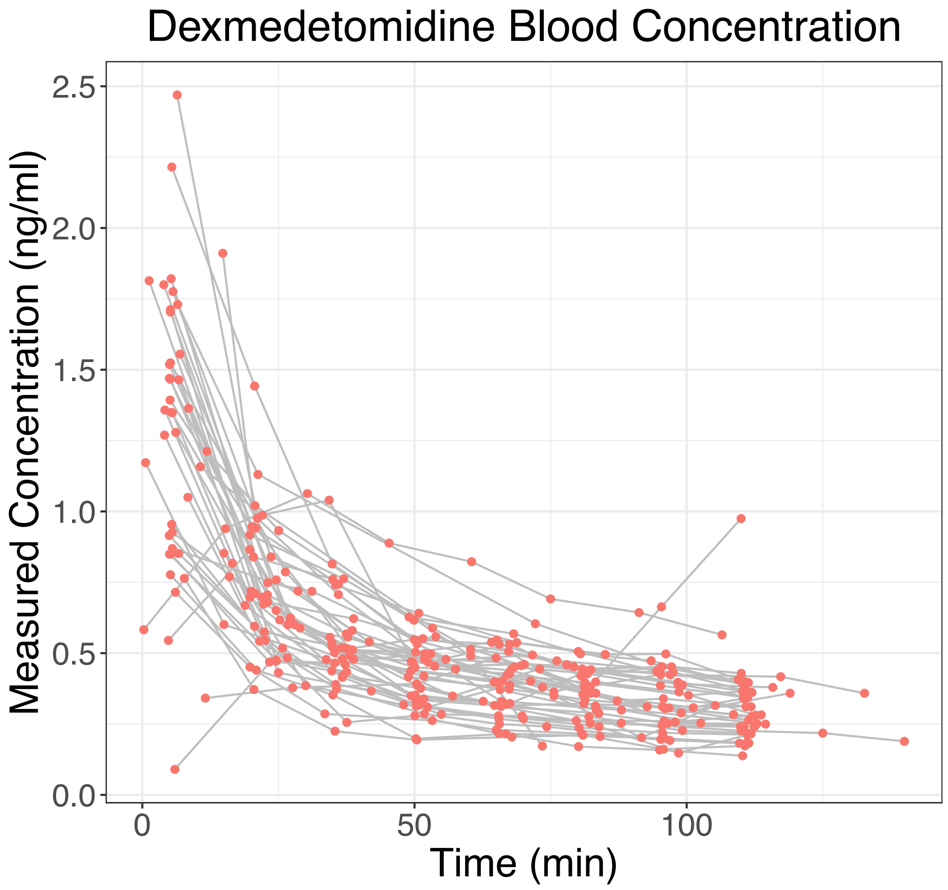

Supplemental Figure 1. Measured concentrations of dexmedetomidine versus time.

Lines connect individual data time points to denote individual patients.

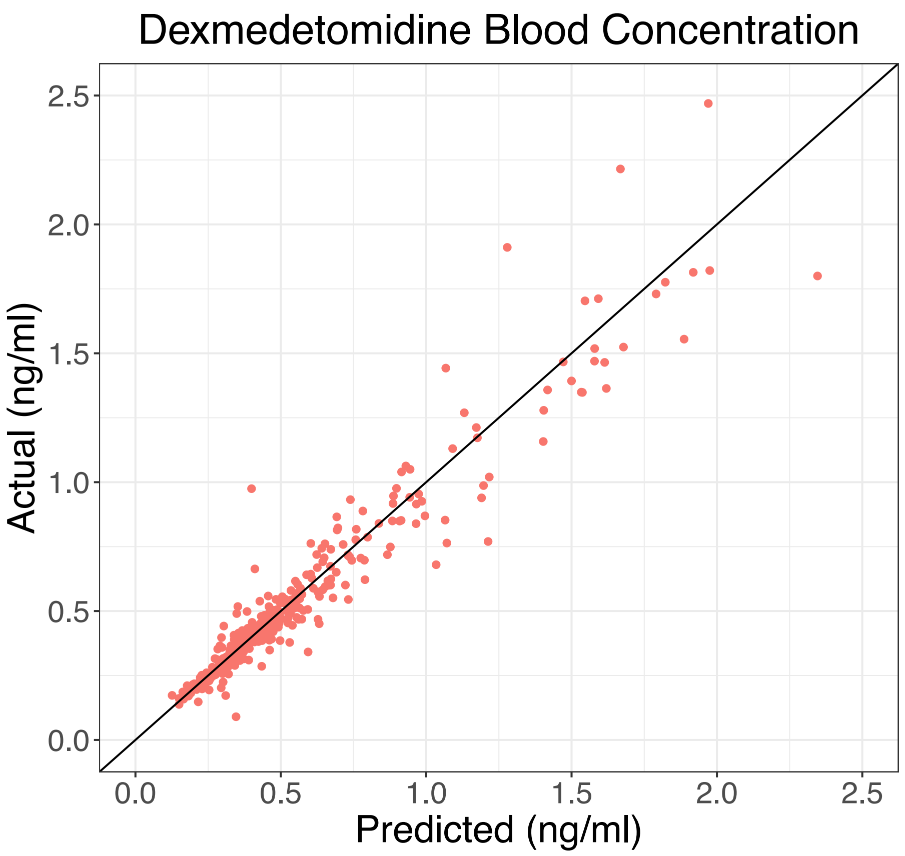

Supplemental Figure 2. Actual versus predicted dexmedetomidine blood concentration based on our pharmacokinetic model.
